# Analyzing Online Conversations on Reddit: A Study of Stress and Anxiety Through Topic Modeling and Sentiment Analysis

**DOI:** 10.7759/cureus.69030

**Published:** 2024-09-09

**Authors:** Rosamma KS

**Affiliations:** 1 Department of Computer Applications, Marian College Kuttikkanam, Kuttikkanam, IND

**Keywords:** latent dirichlet allocation (lda), natural language processing (nlp), reddit, sentiment analysis, stress and anxiety

## Abstract

This study analyses the topic of stress and anxiety in 3,765 Reddit posts to determine key themes and emotional undertones using natural language processing (NLP) techniques. Five major category topics are identified from the posts using the latent Dirichlet allocation (LDA) algorithm. The topics identified are general discontent and lack of direction; panic and anxiety attacks; physical symptoms of anxiety, stress, and mental health concerns; and seeking help for anxiety. Sentiment analysis with the help of TextBlob showed a neutral score, for the most part: an average polarity score of 0.009 and a subjectivity score of 0.494. Several kinds of visualizations, including word clouds, bar charts, and pie charts, have been used to show the distribution and importance of these topics. These findings underscore the important role played by online communities in extending their support to those in distress because of mental health problems. This information is very important to mental health professionals and researchers. This study shows the effectiveness of using a combination of topic modeling and sentiment analysis to identify problems related to mental health discussed on social media. These results direct the possibilities for future research in using advanced NLP techniques and expanding to larger datasets.

## Introduction

Social media has dramatically changed how people communicate and share their experiences, from help-seeking on multiple platforms to mental health support. One of the main social media sites is Reddit, which has numerous sub-communities or subreddits. In these, there exists a special place for raising and discussing personal challenges such as stress and anxiety. The anonymity of Reddit enables more honest sharing. For this reason, it provides an important source of knowledge about online discourse concerning mental illness [1].

Stress and anxiety disorders rank very high on the list of common mental health problems in the world. The quality of life is severely hampered in the sufferer. It goes with symptoms such as physical health complications, decreased functioning, and a general state of well-being low in self-experience. The knowledge of themes and emotional tones in the social media discussions on stress and anxiety helps bring several insights into the experiences of suffering people.

Several studies have been done to research the possibility of analyzing data from social media for tracing mental illnesses. For instance, it was demonstrated that posts on Twitter could be used to predict future levels of depression. De Choudhury et al. [2] Coppersmith et al. [3] expanded on this and utilized Twitter data in identifying signals related to mental health conditions that included depression and bipolar disorder. Reddit has also been a platform for several mental health studies. Researchers have conducted machine learning-based studies on Reddit posts to retrieve meaningful insights [4,5].

In this light, this work examines 3,765 posts from Reddit under which the discussion of the two problems was captured: stress and anxiety. Latent Dirichlet allocation (LDA) [6] is used for topic modeling. Sentiment analysis [7] uses the TextBlob method [8] to gauge the emotional tone of conversations. Such an approach in combination sheds light not only on the content but also on the sentiment of the discussions taking place on Reddit regarding stress and anxiety. The sentiment analysis demonstrates a mainly neutral sentiment in the posts, with a mean polarity score of 0.009 and a mean subjectivity score of 0.494. This reveals diversified emotional expressions on the part of the community: from negativity to positivity in sentiments and from objectivity to subjectivity [9] in viewpoints.

This research assesses these scant concerns at the helm of the stress- and anxiety-related discussions in Reddit such as directed at the main topics, the dominant sentiments, and how such insights could help practitioners and researchers in mental health. This work is important in that it could provide insight into online discussion of mental health issues to be able to comprehend common concerns, as well as the emotional state of persons being stressed and anxious. The results can also provide input into developing targeted interventions and support systems, which are working to improve mental health monitoring through social media data as a scalable tool to identify trends and emerging issues in real time. In addition to that, this study is the foundation for forthcoming research using advanced techniques of natural language processing (NLP) in measuring mental health trends across other social media platforms. The current study, however, goes on to answer these questions and interpret the findings, thus trying to contribute meaningfully toward the growing literature on mental health and social media for the development of efficacious interventions and support mechanisms at large.

## Materials and methods

Dataset

This study used a dataset of 4,000 Reddit entries about stress and anxiety. These dialogues serve as a rich source for textual analysis, giving insight into the popularity and context of stress and anxiety in online conversations. The dataset is freely available on Kaggle, in the form of a .csv file with two columns [10]. It is updated in Kaggle under the Massachusetts Institute of Technology (MIT) license in July 2024. The first column title is Text and contains the Reddit post contents as values. The second column title is Is_stressed/anxious, and it indicates the presence and absence of stress contents in posts with the values 1 and 0, respectively. After data cleaning and pre-processing steps, the dataset contained 3,765 posts labeled with "1".

Experimental setup

The experiments were held in the Google Colab [11] interface, where Python 3.0 provided a powerful and flexible platform for machine learning and NLP tasks. Table 1 outlines the libraries used in the study, each serving a specific function. Pandas is employed for data manipulation and analysis, while Numpy handles numerical computations. The natural language toolkit (NLTK) is used for text preprocessing tasks such as tokenization and stopword removal. Scikit-learn facilitates machine learning tasks, including vectorization and LDA modeling. TextBlob is utilized for sentiment analysis, and Gensim is used for topic modeling and coherence score calculation. Matplotlib and Seaborn are applied for data visualization, and WordCloud generates visual word clouds to represent text data graphically.

**Table 1:** Python libraries used for the pre-processing and analysis of the dataset

Library	Usage
Pandas	Data manipulation and analysis.
Numpy	Numerical computations.
NLTK (Natural Language Toolkit)	Text preprocessing, including tokenization and stopwords removal.
Scikit-learn	Machine learning tasks, including vectorization and LDA modeling
TextBlob	Sentiment analysis
Gensim	Topic modeling and coherence score calculation
Matplotlib and Seaborn	Data visualization
WordCloud	Word cloud generation.

Data preprocessing

The text pre-processing [12] steps employed in this study ensure that the data are thoroughly cleaned and standardized for effective application of NLP techniques such as topic modeling and sentiment analysis. Initially, posts with missing text content were removed to avoid incomplete data that could lead to inaccuracies, as missing values can corrupt data and skew results. The text was then converted to lowercase for consistency, and noisy elements such as punctuation, URLs, user mentions, and non-alphabetic characters were eliminated to focus on the meaningful content of the posts. Next, the cleaned text was tokenized, breaking it down into individual words or tokens, which are essential for extracting meaningful patterns and features [13]. Stop words, commonly used words that do not contribute significant meaning, were removed to avoid distraction from more informative terms [14]. Finally, lemmatization was applied, reducing words to their base or root form, which allows different forms of a word to be treated as a single item, thereby enhancing the coherence of the analysis [15]. Together, these pre-processing steps ensured that the text data were optimally prepared for advanced NLP techniques, enabling accurate topic modeling and sentiment analysis.

Topic modeling

The LDA method was employed to uncover latent thematic structures within the text data, leveraging its role as a generative probabilistic model, which is one of the most widely used techniques for topic discovery in NLP. The process began with vectorization, where the cleaned text was transformed into a numerical format using the CountVectorizer from scikit-learn [16]. This step converted the text into a matrix of raw counts, denoted as X∈R ^m×n^, where m represents the number of documents and n the vocabulary size. The matrix X_ij​ _represents the count of word j in document i, which allowed the LDA model to determine the word distribution across the dataset.

Next, the model definition step involved defining an LDA model to extract five distinct topics, a number determined through exploratory experiments that optimized the model's coherence score C_k_ calculated as the average pairwise similarity between the top words of a topic. Coherence score is a crucial metric in evaluating the quality of topics, and several configurations were tested to select the most coherent set of topics [17].

The model fitting phase entailed fitting the LDA model to the vectorized text data. This process involves iterating over the data to maximize the likelihood p(W∣T) of the observed word distribution given the topics, where W represents the words and T the topics. Specifically, LDA assumes that each document d is generated by a mixture of topics, where each topic k is characterized by a distribution over words. The model adjusts the distribution ϕ_k _of words over topics k to maximize this likelihood, effectively capturing the thematic structure inherent in the dataset [18].

Finally, in the topic modeling interpretation stage, the top words under each topic were manually reviewed, and the primary themes were defined. This critical step involved interpreting the ϕ_k _distributions to understand the nature of the discussions, identifying the prominent issues within the dataset, and labeling the topics according to the most representative words. This interpretation is key to translating the mathematical outcomes of the LDA model into meaningful insights that reflect the core themes and concerns present in the text data.

Sentiment analysis

The sentiment analysis for reading emotional tone was conducted with the use of TextBlob. TextBlob is a Python library that enables a simple API to dive into common NLP, including sentiment analysis. It was done in two steps. The first step is used to find the sentiment polarity and subjectivity, and the scores of polarity and subjectivity were calculated for each post. The polarity scores range from -1 (very negative) to +1 (very positive), thus denoting sentiment, while subjectivity scores denote how much of the text is based on personal opinion rather than facts [19]. The second step analyzed the sentiment polarity distribution graph, visualizing the overall emotional tone of the posts. This plot would help in understanding the general mood and feelings expressed in the dataset.

Evaluation metrics

In this study, the coherence score [20] was the main metric to assess the LDA model. Coherence scores relate to measuring the kind of semantic similarity that is within a given topic, swearing their importance in terms of scoring quality and readability of the said topics. Higher coherence scores show the topics to be coherent and interpretable, thus ensuring reliability for results in topic modeling.

## Results

Insights from the dataset

**Figure 1:**
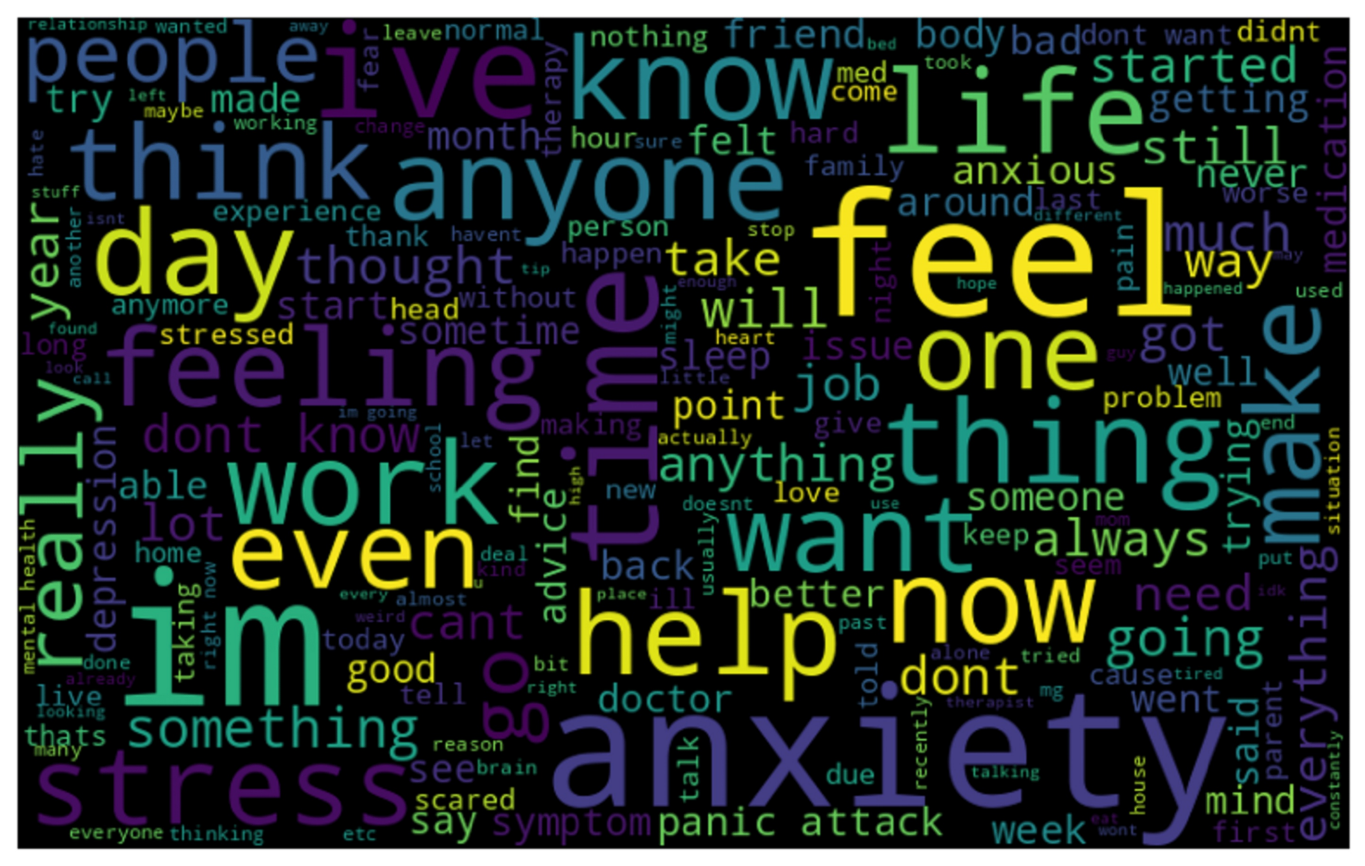
Word cloud representation of the dataset

The Reddit dataset's word cloud as given in Figure 1 reveals the most common terms in conversations about stress and anxiety. Frequently mentioned words such as "feel," "anxiety," "im," "one," "thing," "life," "day," "work," "help," and "know" suggest that these are key topics. Users often share their emotional experiences, with words such as "feeling," "stressed," "anxious," "depression," "scared," "panic," "attack," and "symptoms" being prevalent. The appearance of words such as "help," "advice," "therapy," and "doctor" shows the community's emphasis on seeking support and professional assistance. Discussions frequently involve everyday activities and personal stories, indicated by words such as "work," "job," "home," "school," "sleep," "live," and "relationship." Time-related terms such as "day," "month," "year," and "time," and expressions of intensity such as "really," "much," "lot," and "hard" emphasize the duration and impact of stress and anxiety. Words indicating action such as "take," "make," "want," "will," "try," "doing," and "going" suggest efforts to handle these feelings, while terms such as "advice," "help," "know," and "think" reflect a strong exchange of support and information among users. This word cloud captures the main themes and concerns of those discussing stress and anxiety on Reddit.

**Figure 2:**
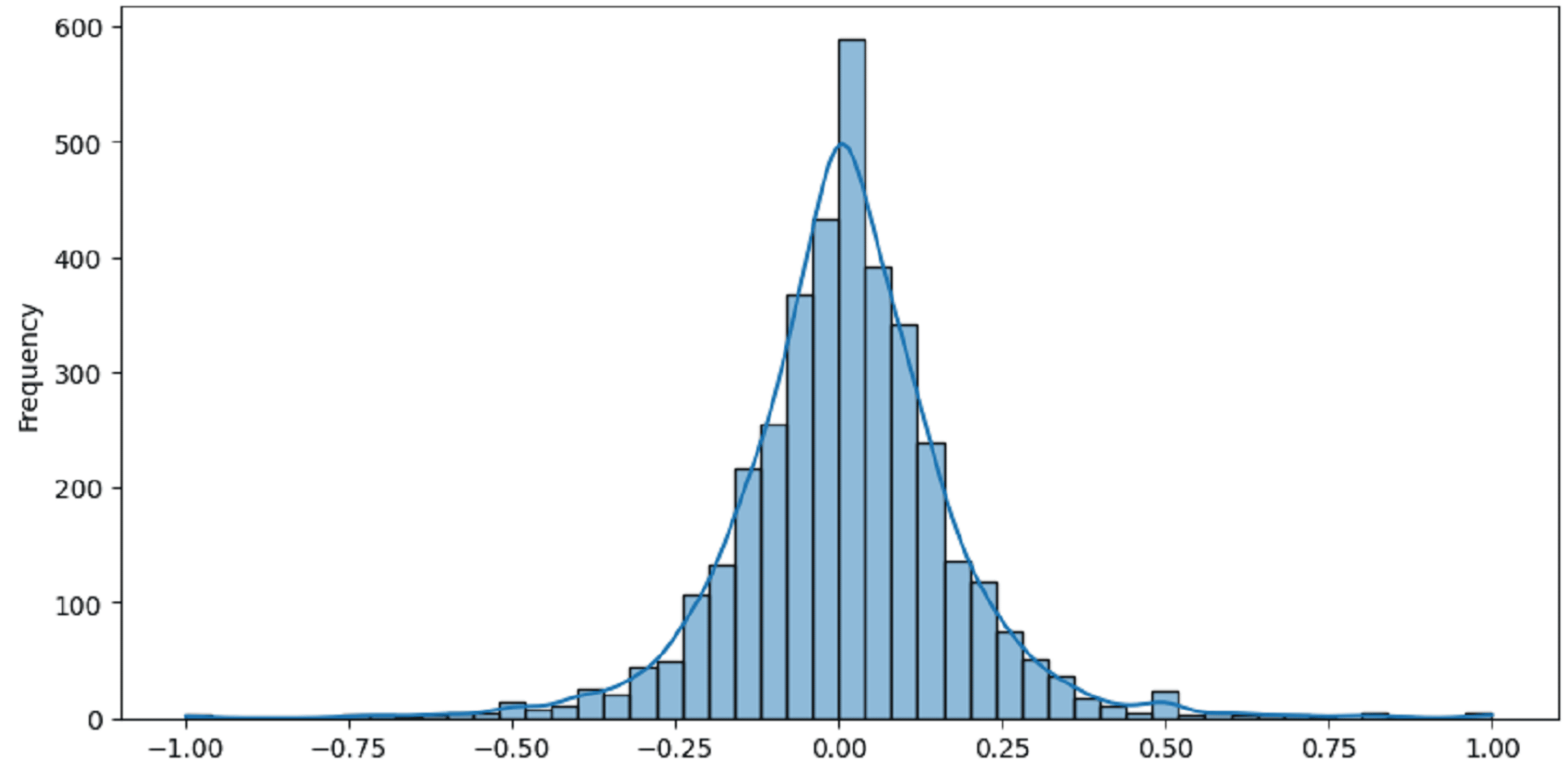
Sentiment polarity distribution of the dataset

The histogram in Figure 2 shows the sentiment polarity distribution. It displays how frequently Reddit posts fall into various sentiment scores, which range from -1 (very negative) to +1 (very positive), with scores near 0 indicating neutral sentiment. The highest concentration of posts is found around the 0 polarity score, meaning most posts are neutral, suggesting that users often share their experiences and opinions in a balanced way without strong emotional extremes. The distribution resembles a bell curve centered around 0, with fewer posts exhibiting extreme positive or negative sentiments. Although there are significant numbers of posts with negative sentiment scores, which indicate users discussing negative experiences and emotions related to stress and anxiety, there are also some posts with positive sentiment scores, though these are less common. The distribution is slightly right-skewed, indicating a slight preference for more positive sentiments over negative ones, but it still covers a wide range of sentiments. This overall balance in sentiment suggests that while many users are sharing neutral or factual content, a notable number are also expressing their emotional challenges and positive experiences, providing insights into the community's general emotional tone.

Topic modeling

**Figure 3:**
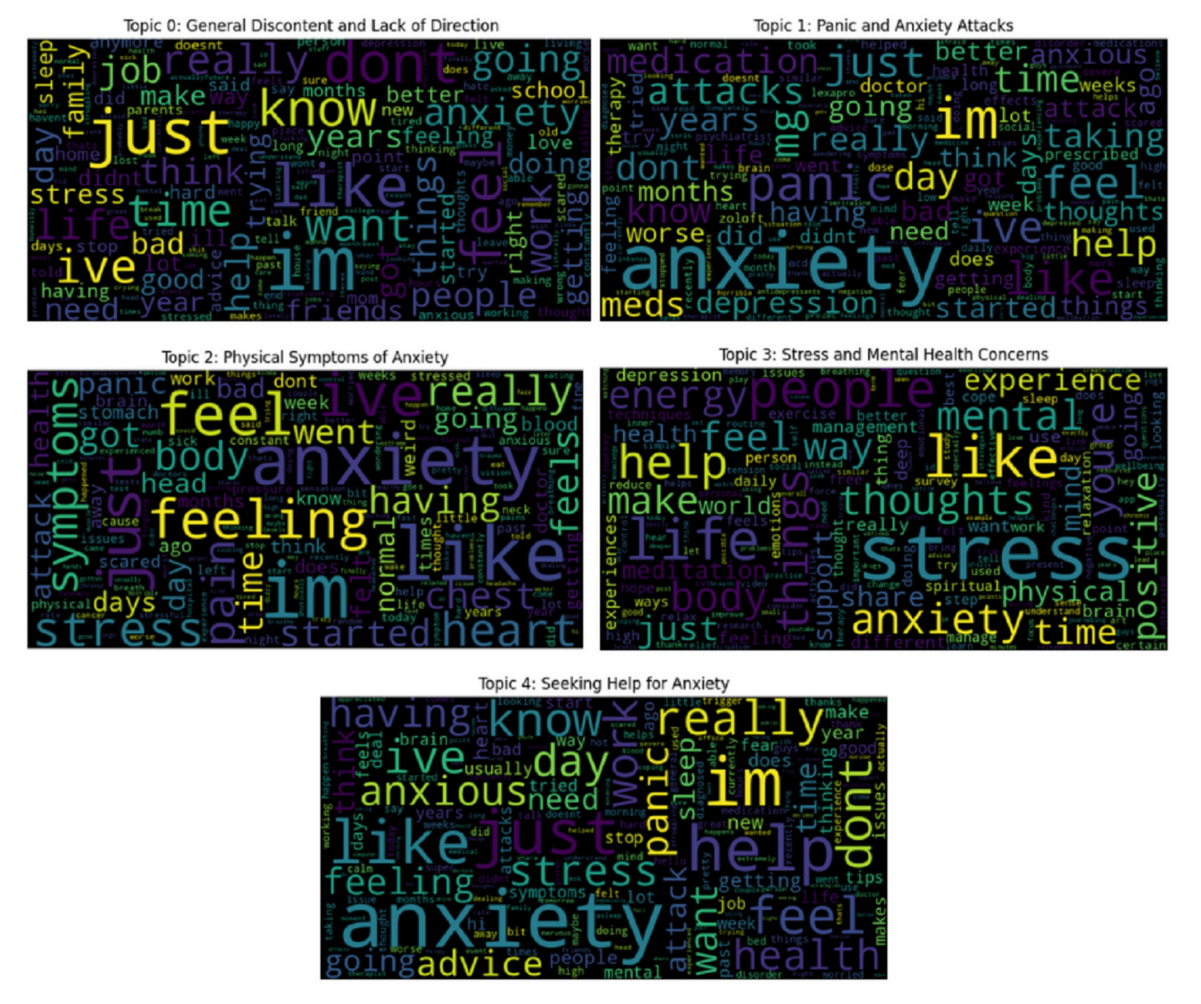
Topic-wise word cloud representation

To uncover the main themes within Reddit posts, utilized the LDA algorithm, which identified five unique topics. Each topic was defined by its most representative words, allowing us to understand the themes. The LDA algorithm generated the topic names as Topic 0, Topic 1 up to Topic 4. However, by using the frequent keywords of each topic, the following topic names are derived manually. For Topic 0, the identified category is general discontent and lack of direction with the following top words: im, just, like, dont, feel, know, ive, time, want, and life. The second topic category is panic and anxiety attacks with the following top words: anxiety, im, panic, like, feel, ive, just, mg, attacks, and day. The third category name identified for Topic 2 is physical symptoms of anxiety with the following keywords: im, like, anxiety, ive, just, feel, feeling, stress, heart, and symptoms. Topic 3 is named with the title stress and mental health concerns since it contains the relevant keywords such as stress, like, life, people, things, help, anxiety, thoughts, mental, and body. Similarly, Topic 4 is named with the title seeking help for anxiety since the top words are anxiety, im, help, just, like, feel, know, dont, really, and I've. These topics emphasize the main areas of concern and discussion among Reddit users who post about stress and anxiety. The word cloud representations in Figure 3 illustrate the same, showcasing each category and the top words within each category.

**Figure 4:**
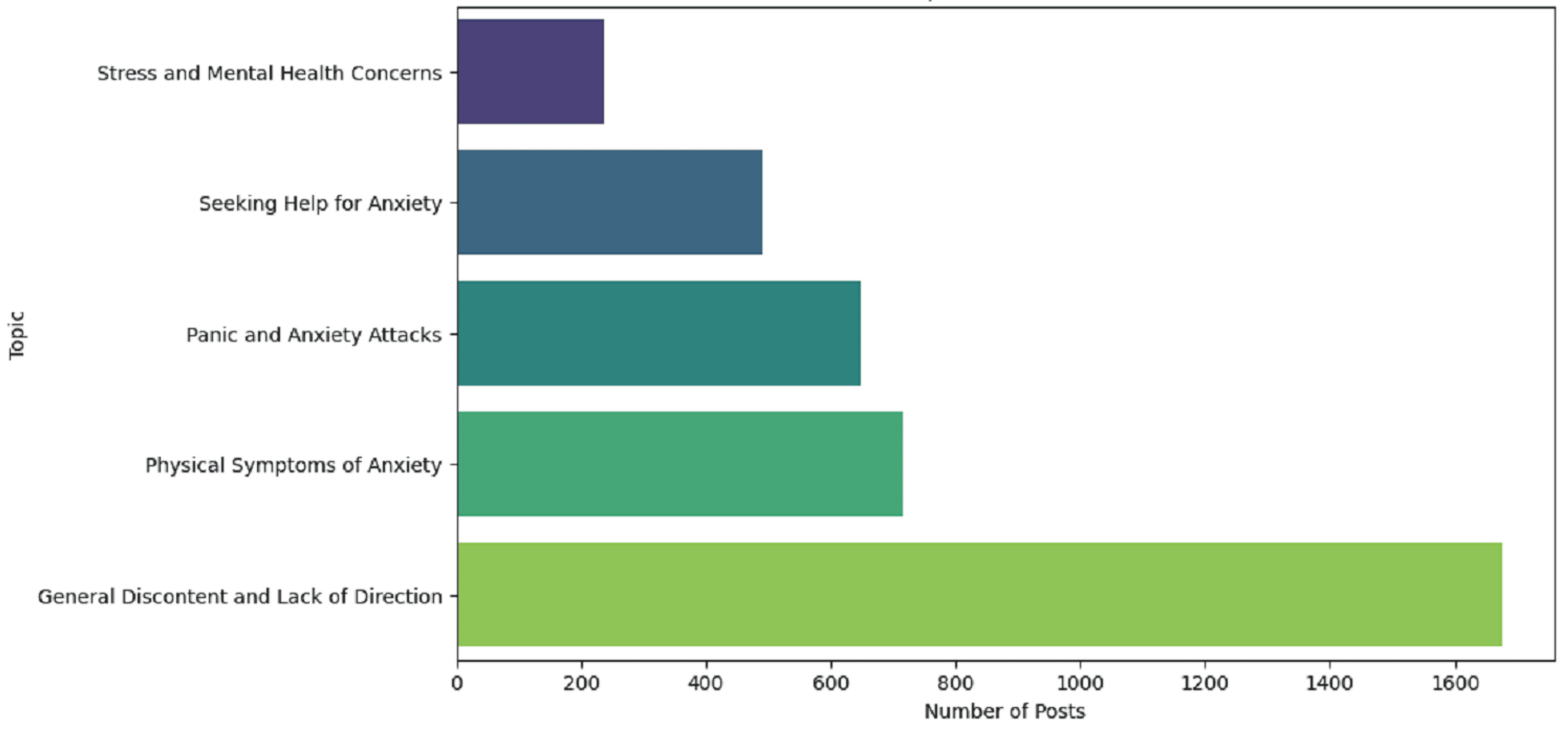
Distribution of topics in stressed or anxious posts

Figure 4 demonstrates the distribution of posts across the five identified topics related to stress and anxiety on Reddit. The topic "general discontent and lack of direction" is the most frequent, with over 1,600 posts, suggesting that many users are talking about feelings of being lost or unhappy with their lives. "Physical symptoms of anxiety" comes second, with about 800 posts, indicating common discussions about the physical effects of anxiety such as heart palpitations or sweating. "Panic and anxiety attacks" also shows a notable number of posts, reflecting that users often share experiences related to sudden anxiety episodes. "Seeking help for anxiety" is another significant topic, with users discussing their attempts to find support and treatment for their anxiety. Lastly, "Stress and mental health concerns" is the least discussed topic but still important, with users talking about broader mental health issues and the impact of stress on their well-being. This distribution underscores the diverse concerns of Reddit users dealing with stress and anxiety, ranging from general dissatisfaction and physical symptoms to seeking help and addressing mental health issues.

**Figure 5:**
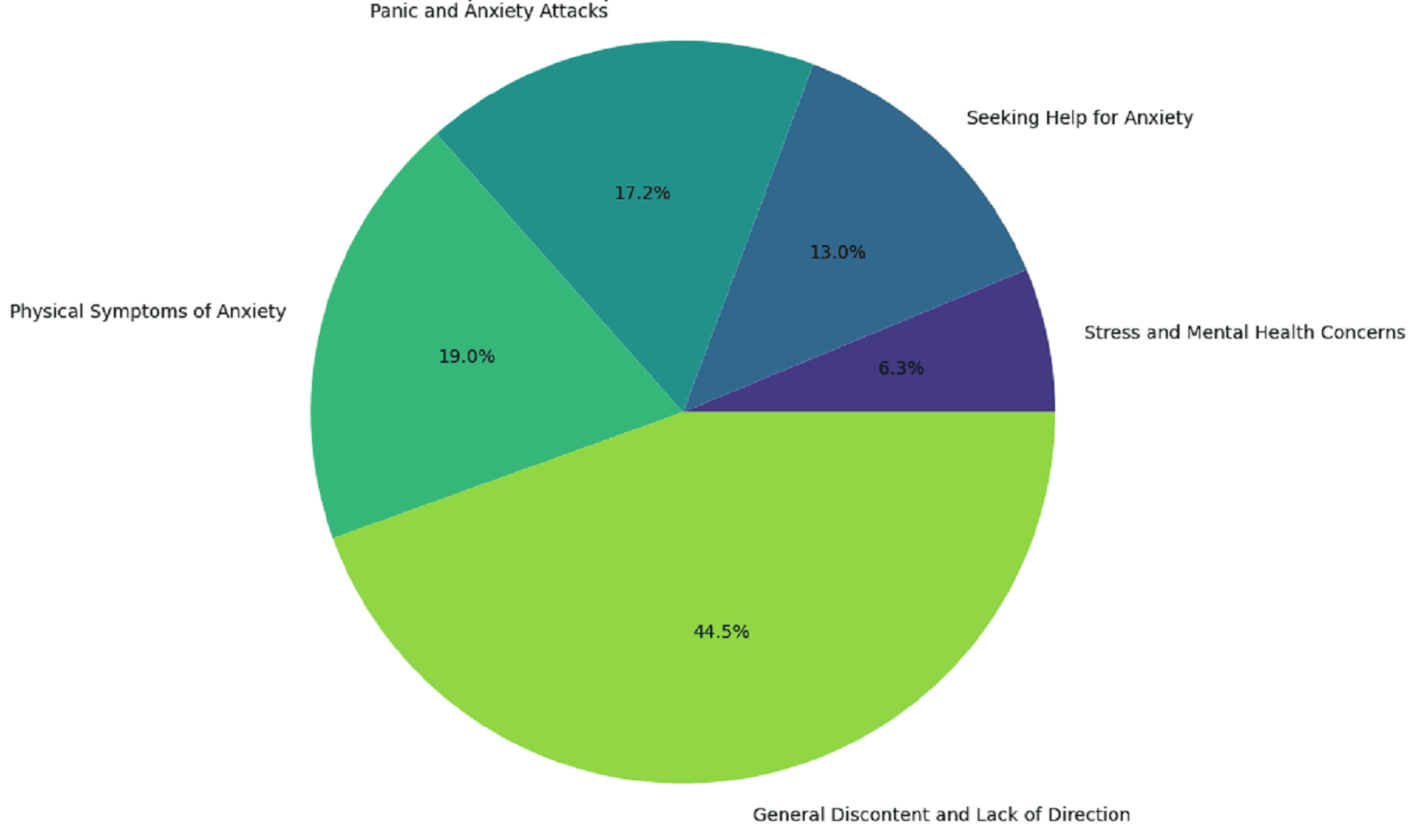
Proportion of topics in stressed or anxious posts

The pie chart in Figure 5 illustrates the proportion of topics discussed in Reddit posts about stress and anxiety. "General discontent and lack of direction" is the most prevalent topic, making up 44.5% of the posts, indicating widespread feelings of dissatisfaction and aimlessness. "Physical symptoms of anxiety" constitutes 19.0% of the posts, highlighting common physical manifestations of anxiety. "Panic and anxiety attacks" represent 17.2%, reflecting significant concern over acute anxiety episodes. "Seeking help for anxiety" accounts for 13.0% of the posts, showing active discussions on seeking support and treatment. Lastly, "stress and mental health concerns" is the least discussed topic at 6.3%, but still an important area of conversation. This proportional distribution emphasizes the variety of issues related to stress and anxiety that users discuss on Reddit.

**Figure 6:**
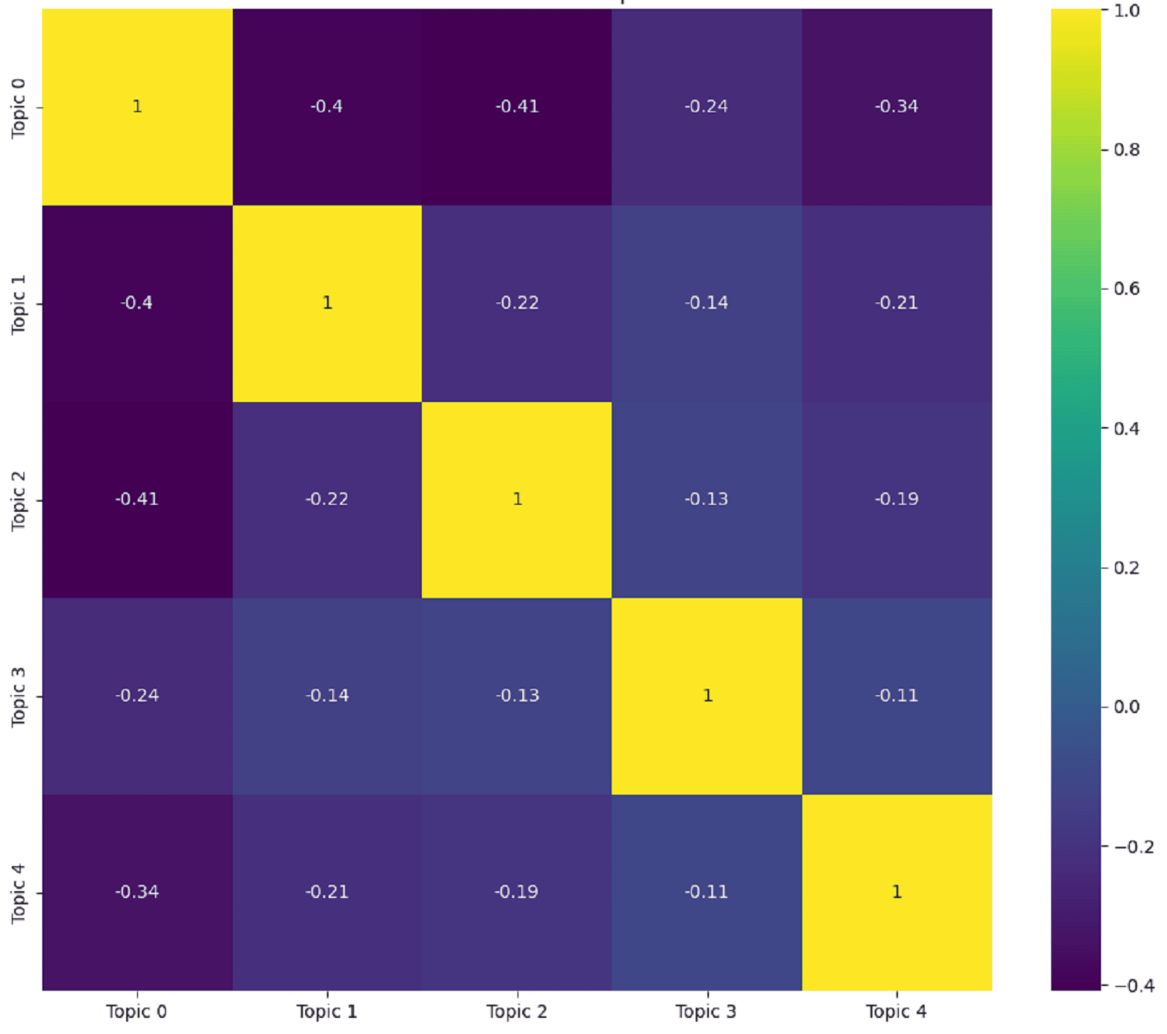
Correlation matrix of topics

The correlation matrix of topics, as given in Figure 6, visualizes the relationships between the identified themes in Reddit posts about stress and anxiety. Each cell in the matrix shows the correlation coefficient between two topics, ranging from -1 to 1. A value near 1 indicates a strong positive correlation, meaning the topics often appear together in the same posts, while a value near -1 indicates a strong negative correlation, meaning the topics seldom appear together. The matrix reveals several key correlations. Topic 0 (general discontent and lack of direction) exhibits moderate negative correlations with all other topics, especially with Topic 2 (physical symptoms of anxiety) (-0.41) and Topic 1 (panic and anxiety attacks) (-0.40). This indicates that discussions about general discontent are often separate from those about specific anxiety symptoms or panic attacks. Topic 1 (panic and anxiety attacks) shows a weaker negative correlation with Topic 4 (seeking help for anxiety) (-0.21) and Topic 3 (stress and mental health concerns) (-0.14), indicating some degree of separation between these themes. Topic 2 (physical symptoms of anxiety) also displays negative correlations with other topics, emphasizing that discussions focusing on physical symptoms are somewhat distinct from broader mental health concerns or seeking help. Topic 3 (stress and mental health concerns) and Topic 4 (seeking help for anxiety) have the least negative correlations with other topics, suggesting these discussions might occasionally overlap with other themes. Overall, the negative correlations imply that each topic is usually discussed in isolation rather than in conjunction with other themes. This separation suggests that users are likely to focus on specific aspects of their stress and anxiety experiences in each post, rather than addressing multiple themes simultaneously.

Linguistic feature analysis

**Figure 7:**
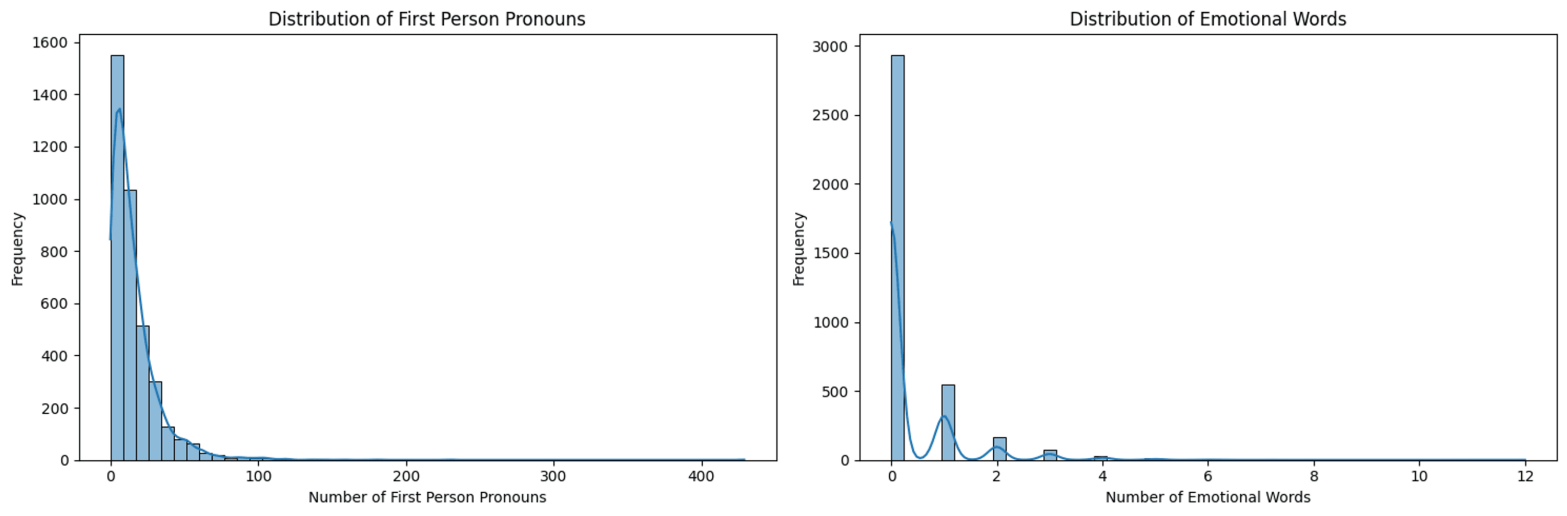
Distribution of first-person pronouns and emotional words

A linguistic feature analysis of the dataset was also done to reveal the common linguistic trends in the posts. The two histograms given in Figure 7 show the distribution of first-person pronouns and emotional words in Reddit posts about stress and anxiety. The left histogram indicates that most posts contain a relatively low number of first-person pronouns, with the highest frequency observed between 0 and 20 pronouns, and very few posts containing more than 50. This implies that users often share personal experiences concisely. The right histogram shows that the majority of posts contain few emotional words, with the highest frequency at 0-1 emotional words per post, and a sharp decrease in frequency as the number of emotional words increases. This suggests that users frequently discuss stress and anxiety with minimal use of overt emotional language. These findings are significant in understanding how users convey their experiences on social media. The tendency to use concise and less emotionally charged language indicates a preference for descriptive narratives over emotional ones. This insight can aid mental health professionals in tailoring their approaches when interacting with individuals on online platforms, highlighting the importance of recognizing the subtleties in how people express their mental health challenges.

**Figure 8:**
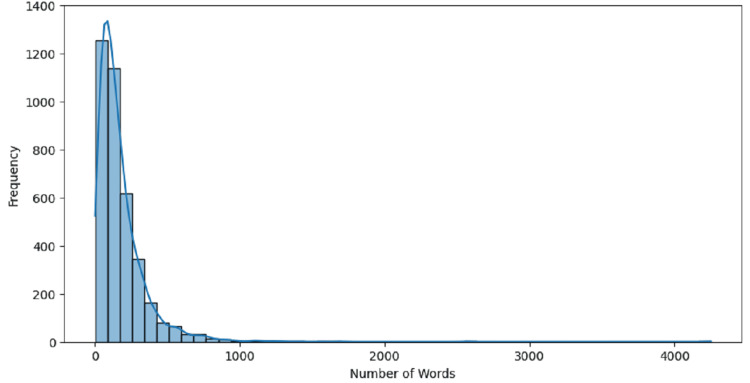
Distribution of post lengths

The histogram in Figure 8 shows post lengths in Reddit discussions about stress and anxiety. It indicates that most posts are relatively brief, with the highest frequency found in the range of 0 to 200 words. The distribution sharply declines as the post length increases, with very few posts exceeding 500 words and a negligible number going beyond 1,000 words. This suggests that users generally prefer to share their experiences and concerns through concise messages rather than lengthy narratives. The importance of this finding lies in understanding user behavior on social media platforms. Shorter posts may indicate a preference for brevity and quick exchanges of information and support. For mental health professionals and researchers, this insight can inform the design of interventions and support mechanisms, emphasizing the need for clear, concise communication and potentially offering more bite-sized resources or responses that align with the user's communication style. Additionally, recognizing the brevity of posts can help tailor content moderation and automated support systems to better meet the needs of users who favor short, direct interactions.

Table 2 presents key metrics derived from the analysis of the posts. The average post length is 176.26 words, indicating that users generally share moderately detailed accounts of their experiences. Sentiment polarity, with an average of 0.009765 and a standard deviation of 0.168359, suggests that the overall sentiment of the posts is neutral, with a balanced mix of slightly positive and negative sentiments. The middle 50% of posts have polarity scores between -0.074643 and 0.094444, further emphasizing the neutrality of the discussions. Sentiment subjectivity, with an average of 0.494119 and a standard deviation of 0.148109, indicates that posts are typically balanced between objective information and personal opinions. The median subjectivity score of 0.500000 shows an equal blend of subjective and objective content, reflecting users' tendencies to combine factual descriptions with personal insights. This summary provides a comprehensive overview of the emotional tone and nature of the discussions, highlighting the importance of addressing both informational and emotional needs in mental health support and interventions.

**Table 2:** Key metrics derived from the dataset

Metric	Value
Total Posts	3,765
Average Post Length	176.26 words
Sentiment Polarity	
- Count	3,765
- Mean	0.009765
- Standard Deviation	0.168359
- Minimum	-1.000000
- 25th Percentile	-0.074643
- Median	0.004896
- 75th Percentile	0.094444
- Maximum	1.000000
Sentiment Subjectivity	
- Count	3,765
- Mean	0.494119
- Standard Deviation	0.148109
- Minimum	0.000000
- 25th Percentile	0.424281
- Median	0.500000
- 75th Percentile	0.572944
- Maximum	1.000000

## Discussion

The analysis of the dataset reveals considerable insight into the nature of discussion threads on Reddit related to stress and anxiety. It clearly demonstrates that most of the posts remain neutral in terms of sentiment polarity distribution, which means users tend to present their ideas and experiences in a quite balanced way. This agrees with previous research that has returned results showing online mental health communities often contain very wide ranges of emotional expression, though neutral sentiments usually predominate [21]. On the other hand, the presence of both negative and positive polarities underlines the variety of emotional expressions within the community. This balanced sentiment distribution underlines, for mental health professionals, equal attention to neutral and charged content in support strategies [22]. This topic modeling across Reddit posts will help in understanding what kinds of discussions are ongoing around stress and anxiety. The strong presence of "general discontent and lack of direction" shows that many users feel lost or poorly satisfied with their lives. The observation corresponds to other studies that demonstrate feelings of general discontent and purposelessness are typical for the human factor in individuals discussing their mental health issues online [23]. Furthermore, high scores on physical symptoms and panic attacks suggest that anxiety is very physical in nature and needs immediate treatment, which corroborates earlier findings that have shown the somatic symptoms most commonly discussed on online forums. Active help-seeking reinforces the community's search for support and treatment.

The correlation matrix indicates that these themes of discussion are relatively distinct, with every post normally focused on certain aspects of the users' experience. This may indicate that people prefer to discuss certain issues in isolation rather than trying to deal with several issues at a time. This is a general trend observed across other online mental health communities as well. Appreciation of this trend could help mental health professionals in terms of better planning and targeting of interventions.

First-person pronouns and emotional language were examined, with the results indicating that the majority of users generally share their experiences in summary form, using very little emotional language. This justifies other studies that have indicated online users are more at ease sharing details of their factual information and personal revelations rather than overtly declaring their emotions [24]. Such awareness may be able to give mental health professionals a guideline for how to engage with users posting online. Moreover, the range of the post lengths suggests that most users prefer concise messages, reflecting a need for brevity and quick information exchange, which is a characteristic observed in similar studies on social media communication [25].

Generally speaking, the findings of this research show that topics and emotional attitudes toward the discussion of stress and anxiety on Reddit are quite diverse. Knowledge of such trends could be used to help mental health professionals and researchers plan more effective interventions and support systems for people who have experienced stress or anxiety. Moreover, such insights can guide future research into the trends of mental health in other social media venues, improving the understanding of online mental health discourses in general.

Apart from all the advantages mentioned above, the results have some limitations. The research only focuses on Reddit, which may not represent all online discussions about stress and anxiety. There is also potential bias from self-reported data and the limitations of text-based analysis in capturing non-verbal cues relevant to mental health. Future research may address these issues to better understand mental health discussions on social media and improve support for those experiencing stress or anxiety in online environments.

## Conclusions

This study goes in-depth into content data from the discussions on stress and anxiety on Reddit, spotting the most relevant topics using advanced natural language processing techniques, such as LDA for topic modeling and TextBlob for sentiment analysis. The study analyzed 3,765 posts and distinguished five themes that emerged: general discontent and lack of direction, panic and anxiety attacks, physical symptoms of anxiety, stress and mental health concerns, and, lastly, seeking help for anxiety. Sentiment analysis of the posts showed that most of the texts were normally toned, with a balanced mixture of plus and minus sentiments from the emotional states of users. It was revealed by the linguistic feature analysis that the tendency of the user is to share his experience briefly without using many emotional words but preferring a more descriptive line of expression.

These findings underline the importance of knowledge about the ways in which users communicate their mental health concerns on social media. Gleaned insights can assist in the development of appropriate mental health interventions and support systems targeting information and affective needs. Mental health professionals and researchers have to become more responsive to interactions with people on online platforms, able to recognize the most dominant themes and the nature of the sentiment, making provisions for more appropriate support and resources.
